# Syntheses, crystal structures and thermal properties of *catena*-poly[cadmium(II)-di-μ-bromido-μ-pyridazine-κ^2^ *N* ^1^:*N* ^2^] and *catena*-poly[cadmium(II)-di-μ-iodido-μ-pyridazine-κ^2^ *N* ^1^:*N* ^2^]

**DOI:** 10.1107/S2056989023002001

**Published:** 2023-03-10

**Authors:** Christian Näther, Inke Jess

**Affiliations:** a Institut für Anorganische Chemie, Universität Kiel, Max-Eyth.-Str. 2, 24118 Kiel, Germany; University of Aberdeen, United Kingdom

**Keywords:** synthesis, crystal structure, cadmium halide coordination polymer, chain compound, thermal properties

## Abstract

The reactions of cadmium bromide and cadmium iodide with pyridazine (C_4_H_4_N_2_) in ethanol under solvothermal conditions led to the formation of crystals of [CdBr_2_(pyridazine)]_*n*_ (**1**) and [CdI_2_(pyridazine)]_*n*_ (**2**), which were characterized by single-crystal X-ray diffraction. The asymmetric units of both compounds consist of a cadmium cation located on the inter­section point of a twofold screw axis and a mirror plane (2/*m*), a halide anion that is located on a mirror plane and a pyridazine ligand, with all atoms occupying Wyckoff position 4*e* (*mm*2). These compounds are isotypic and consist of cadmium cations that are octa­hedrally coordinated by four halide anions and two pyridazine ligands and are linked into [100] chains by pairs of μ-1,1-bridging halide anions and bridging pyridazine ligands. In the crystals, the pyridazine ligands of neighboring chains are stacked onto each other, indicating π–π inter­actions. Larger amounts of pure samples can also be obtained by stirring at room-temperature, as proven by powder X-ray diffraction. Measurements using thermogravimetry and differential thermoanalysis (TG-DTA) reveal that upon heating all the pyridiazine ligands are removed in one step, which leads to the formation of CdBr_2_ or CdI_2_.

## Abstract 2

In the crystal structures of the title compounds, the cadmium cations are octa­hedrally coordinated by four halide anions and two pyridazine ligands in a *trans*-Cd*X*_4_N_2_ (*X* = Br, I) arrangement and are linked into chains by the halide anions and the pyridazine ligands.

## 1. Chemical context

Coordination polymers based on transition-metal halides show a versatile structural behavior and can form networks of different dimensionalities (Peng *et al.*, 2010 ▸). This is especially valid for compounds based on Cu^I^, which show different Cu*X* substructures (*X* = Cl, Br, I) such as, for example, dimeric units, chains or layers that can be additionally connected by bridging neutral coligands (Peng *et al.*, 2010 ▸). These compounds are of additional inter­est because of their lumin­escence behavior (Gibbons *et al.*, 2017 ▸; Mensah *et al.*, 2022 ▸). For one particular metal halide and coligand, compounds of different stoichiometry are frequently observed. In most cases they were synthesized in the liquid state, but in some cases the coligand-deficient phases cannot be obtained from solution or are obtained only as mixtures with coligand-rich phases.

We have been inter­ested in the structural properties of such compounds for several years and have found that upon heating most of the coligand-rich compounds lose their co­ligands stepwise and transform into new coligand-deficient compounds that show condensed copper-halide networks (Näther & Jess, 2004 ▸; Näther *et al.*, 2001 ▸, 2007 ▸). The advantage of this method is the fact that this reaction is irreversible, and that the new compounds are obtained in qu­anti­tative yields. Moreover, in some cases, metastable polymorphs or isomers can also be obtained (Näther *et al.*, 2007 ▸) and this method can also be used for the synthesis of new coordination polymers with other bridging anionic ligands such as, for example, thio- or seleno­cyanates (Werner *et al.*, 2015 ▸; Wriedt & Näther, 2010 ▸).

We subsequently found that transition-metal halide compounds with twofold positively charged cations such as Cd^II^ that also show a pronounced structural variability can be obtained by this route (Näther *et al.*, 2017 ▸; Jess *et al.*, 2020 ▸). In most cases, discrete Cd*X*_2_ complexes are observed (Ghanbari *et al.*, 2017 ▸; Liu, 2011 ▸), but these units can also condense into dinuclear (Santra *et al.*, 2016 ▸; Xie *et al.*, 2003 ▸) and tetra­nuclear units (Zhu, 2011 ▸) or polymers (Nezhadali Baghan *et al.*, 2021 ▸; Satoh *et al.*, 2001 ▸), where the latter can be further linked by the coligands into layers (Hu *et al.*, 2009 ▸; Marchetti *et al.*, 2011 ▸).

In this context, we have reported on Cd*X*_2_ coordination polymers with 2-chloro and 2-methyl­pyrazine with the composition Cd*X*_2_(*L*)_2_ with *X* = Cl, Br, I and *L* = 2-chloro or 2-methyl­pyrazine). These compounds consists of Cd*X*_2_ chains in which the Cd cations are linked by two pairs of μ-1,1-bridging halide anions (Näther *et al.*, 2017 ▸). Surprisingly, upon heating, the compounds with 2-chloro­pyrazine lose all the coligands in one single step, whereas decomposition of the 2-methyl­pyrazine compounds leads to the formation of compounds with the composition Cd*X*_2_(2-methyl­pyrazine), in which the CD*X*_2_ chains are linked into layers by the 2-meth­yl­pyrazine ligands. These compounds can also be obtained if the discrete complex CdI_2_(2-methyl­pyrazine)_2_(H_2_O) is thermally decomposed. In further work we investigated similar compounds with 2-cyano­pyrazine as coligand, where we observed a different thermal reactivity as a function of the nature of the halide anions (Jess *et al.*, 2020 ▸).

In the course of our investigations we also became inter­ested in compounds with pyridazine as coligand. A search in the CCDC database revealed that several transition-metal halide coordination compounds with this ligand have already been reported in the literature (see *Database survey*). With cadmium, one compound with the composition CdCl_2_(pyridazine) is reported, in which the Cd^II^ cations are linked by μ-1,1-bridging chloride anions into chains, in which each two Cd^II^ cations are additionally connected by the pyridazine ligands (Pazderski *et al.*, 2004*a* ▸). As this compound is isotypic to many other M*X*_2_(pyridazine) coordination compounds, one can assume that this structure represents a very stable arrangement. On the other hand, compounds with this composition have also been reported with Zn*X*_2_. In contrast to the bromide and iodide compounds, the chloride analog crystallizes in three different modifications, which indicates that the structural behavior also depends on the nature of the halide anion (Bhosekar *et al.*, 2006*a* ▸,*b* ▸; Pazderski *et al.*, 2004*b* ▸; Bhosekar *et al.*, 2007 ▸). Moreover, even if in the majority of compounds Nezhadali acts as a bridging ligands, some examples have been reported in which this ligand is coordinated to metal cations with only one of the two N atoms, thereby forming discrete complexes, which also include transition-metal halide complexes (Handy *et al.*, 2017 ▸; Boeckmann *et al.*, 2011 ▸; Laramée & Hanan, 2014 ▸; Yang, 2017 ▸; Harvey *et al.*, 2004 ▸). 
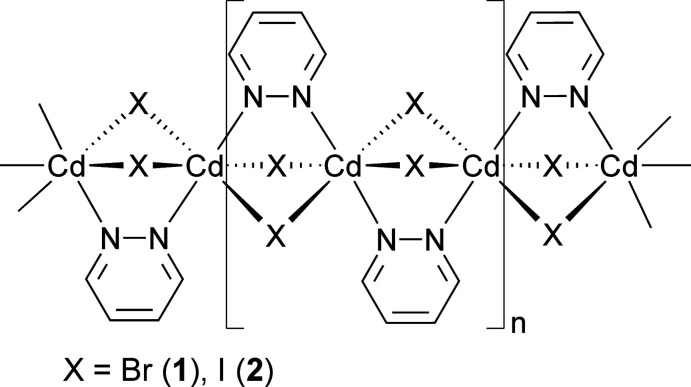


Based on all these findings, we reacted CdBr_2_ and CdI_2_ in different molar ratios with pyridazine in several solvents to investigate whether compounds with a different ratio between Cd*X*_2_ and pyridazine can be prepared, which also might include pyridazine-rich discrete complexes that upon heating might transform into new compounds with a more condensed network. However, independent of the reaction conditions and the stoichiometric ratio, we always obtained the same crystalline phases, as proven by powder X-ray diffraction (PXRD). Crystals of both compounds were obtained at elevated temperatures and structure analysis proves that compounds with the composition CdBr_2_(pyridazine) (**1**) and CdI_2_(pyridazine) (**2**) were obtained. Comparison of the experimental PXRD patterns with those calculated from the results of the structure determinations, prove that both compounds were obtained as pure phases (Figs. S1 and S2). Measurements using thermogravimetry and differential thermoanalysis reveal that both compounds decompose in one step, which is accompanied with an endothermic event in the DTA curve (Figs. S3 and S4). The experimental mass losses of 22.9% for **1** and 18.1% for **2** are in good agreement with those calculated for the removal of one pyridazine ligand (Δ*m*_calc._ = 22.7% for **1** and 17.9% for **2**), indicating that CdBr_2_ and CdI_2_, respectively, have formed.

In this context, it is noted that the formation of a more pyridazine-deficient compound with a more condensed network is not expected, because for *M* ^2+^ cations, the network should be negatively charged. This is impossible in this case, but it is noted that one compound with CdCl_2_ and a more condensed metal–halide network is reported in the literature (Jin *et al.*, 2014 ▸).

## 2. Structural commentary

The reaction of cadmium dibromide or cadmium diiodide with pyridazine leads to the formation of crystals of CdBr_2_(pyridazine) (**1**) and CdI_2_(pyridazine) (**2**). Both compounds are isotypic to their CdCl_2_ analog already reported in the literature (Pazderski *et al.*, 2004*a* ▸). In this context, it is noted that for compound **2** a pseudo-translation along the crystallographic *b*-axis is detected, leading to half of the unit cell and space group *Cmmm* but the refinement clearly shows that the present unit cell and space group is correct (see *Refinement*). Both compounds are also isotypic to a number of other metal–halide coordination polymers, indicating that this is a very stable arrangement (see *Database survey*).

**Figure 1:**
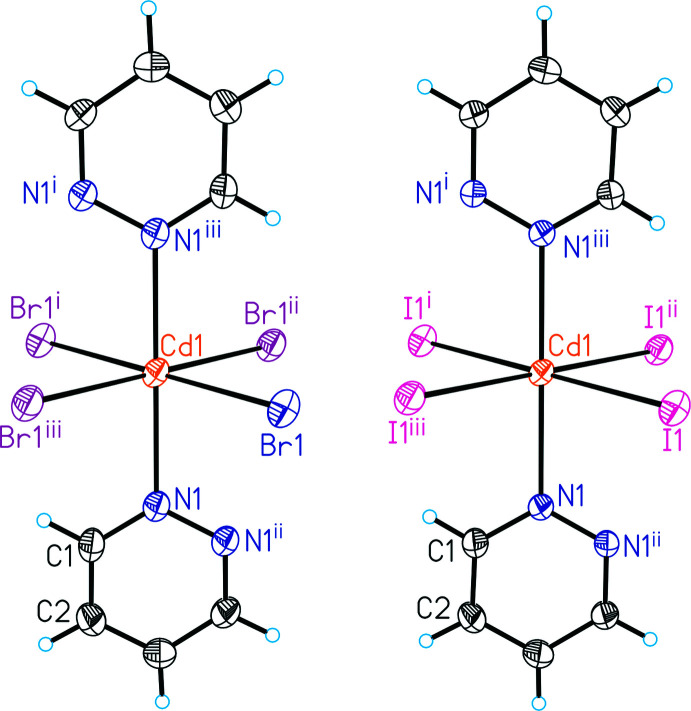
The metal atom polyhedra in **1** (left) and **2** (right) with labeling and displacement ellipsoids drawn at the 50% probability level. Symmetry codes for the generation of equivalent atoms: (i) −*x* + 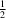 , −*y* + 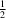 , −*z* + 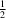 ; (ii) −*x* + 1, −*y* + 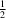 , *z*; (iii) *x* − 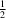 , *y*, −*z* + 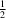 .

**Figure 2:**
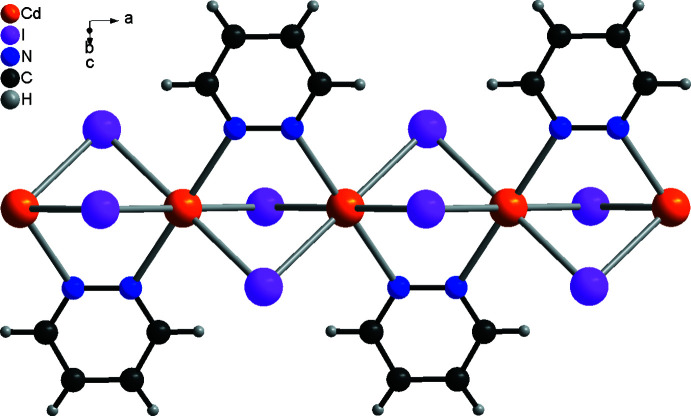
Fragment of a [100] polymeric chain in the crystal structure of **1**.

The asymmetric units of compound **1** and **2** consist of a cadmium cation located on the inter­section point of a twofold screw axis and a mirror plane (Wyckoff site 4*c*, symmetry 2/*m*), as well as a bromide or iodide anion lying on a mirror plane (Wyckoff site 8*h*) and a pyridazine ligand, with all atoms located on Wyckoff position 4*e* (*mm*2) (Fig. 1 ▸). In both compounds, the Cd^II^ cations are octa­hedrally in a *trans*-Cd*X*_4_N_2_ arrangement, coordinated by four halide anions and two pyridazine ligands, and are linked by pairs of μ-1,1-bridg­ing halide anions into chains that propagate in the crystallographic *a*-axis direction (Fig. 2 ▸). The pyridazine ligands also act as bridging ligands, connecting two neighboring Cd^II^ cations (Fig. 2 ▸). Within the chains, all of the pyridazine ligands are coplanar. (Fig. 2 ▸).

The Cd—N bond lengths to the pyridazine ligand are slightly longer in the iodide compound **2** compared to compound **1**, which might be traced back to some crowding of the bulky iodide anion. In agreement, this distance is the shortest in the corresponding chloride compound (Pazderski *et al.*, 2004*a* ▸) reported in the literature (Tables 1 ▸ and 2 ▸). The N—Cd—Br and N—Cd—I bond angles are comparable, which is also valid for that in the chloride compound (Pazderski *et al.*, 2004*a* ▸). As expected, the intra­chain Cd⋯Cd distance increases from Cl [Cd⋯Cd = 3.5280 (5) Å], to Br [Cd⋯Cd = 3.6270 (3) Å] to I [Cd⋯Cd = 3.7870 (3) Å].

## 3. Supra­molecular features

**Figure 3:**
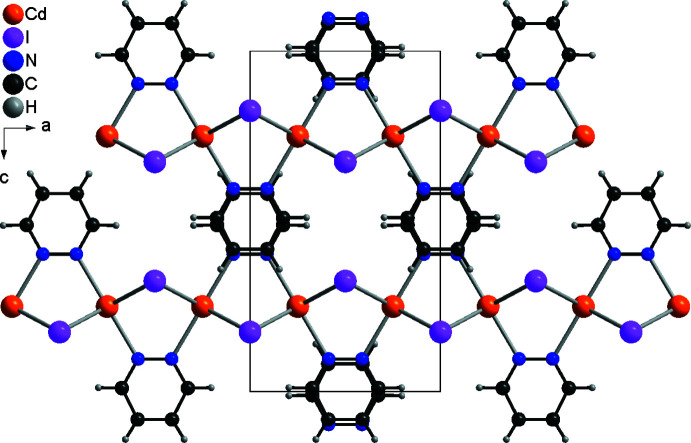
Arrangement of the chains in the crystal structure of **1** in a view along the crystallographic *b*-axis direction.

**Figure 4:**
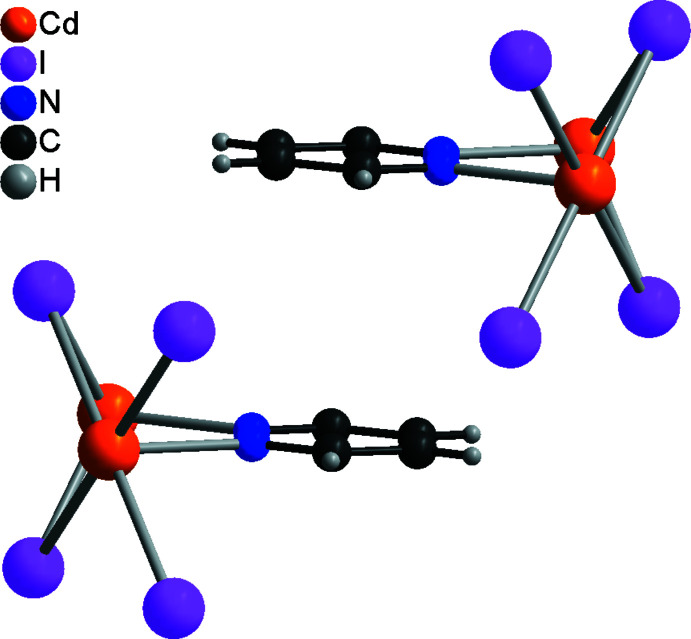
Arrangement of neighboring pyridazine rings in **1** showing π–π stacking inter­actions.

In the crystal structures of **1** and **2**, the chains extend in the crystallographic *a*-axis direction (Fig. 2 ▸). Neighboring chains are arranged in such a way that the pyridazine ligands are perfectly stacked onto each other into columns that propagate along the crystallographic *b*-axis direction (Fig. 3 ▸). The angle between two neighboring pyridazine ligands is 180° in both compounds, which is also valid for the chloride analog (Pazderski *et al.*, 2004*a* ▸). The distance between the centroids of adjacent pyridazine rings is 3.724 Å for the chloride, 3.8623 (1) Å (slippage = 0.095 Å) for the bromide and 4.1551 (1) Å (0.226 Å) for the iodide, consistent with π–π inter­actions (Fig. 4 ▸), although they must be weak for the iodide. There are no directional inter­molecular inter­actions such as inter­molecular C—H⋯*X* hydrogen bonding. As mentioned above, this structure type is common for the majority of transition-metal pyridazine coordination compounds with such a metal-to-pyridazine ratio, indicating that π–π inter­actions might also be responsible for this obviously very stable arrangement.

## 4. Database survey

A search in the CCDC database (version 5.43, last update November 2022; Groom *et al.*, 2016 ▸) revealed that some compounds with the general composition *MX*_2_(pyridazine) (*M* = transition metal and *X* = halide anion) have already been reported in the literature. The compounds with NiCl_2_ (CSD refcode POPCIG) and NiBr_2_ (POPCOM) were structurally characterized by Rietveld refinements using laboratory X-ray powder diffraction data and are isotypic to the title compounds (Masciocchi *et al.*, 1994 ▸). In this contribution, the compounds with Mn, Fe, Co, Cu and Zn with chloride and bromide as anions were also synthesized, and their lattice parameters determined from their powder patterns, indicating that the compounds with Mn, Fe and Co are isotypic to the Ni compound, which is not the case for the compounds with Cu and Zn (Masciocchi *et al.*, 1994 ▸). The compounds *M*Cl_2_(pyridazine) with Mn (LANJEQ) and Fe (LANJAM) were later determined by single-crystal X-ray diffraction, which definitely proves that they crystallize in space group *Immm* (Yi *et al.*, 2002 ▸).

In this context it is noted that three compounds containing diamagnetic Zn^II^ cations have been reported, which consist of discrete complexes with a tetra­hedral coordination, *viz*. ZnI_2_(pyridazine)_2_ (MENSUU; Bhosekar *et al.*, 2006*a* ▸), ZnBr_2_(pyridazine)_2_ (VEMBEV; Bhosekar *et al.*, 2006*b* ▸) and three modifications of CuCl_2_(pyridazine)_2_ (YAFYOU, YAFYOU01, YAFYOU02 and YAFYOU03; Pazderski *et al.*, 2004*b* ▸ and Bhosekar *et al.*, 2007 ▸). Surprisingly, none of the different forms are isotypic to the chloride and bromide compounds reported by Masciocchi *et al.* (1994 ▸) based on XRPD patterns.

With Cu^II^ cations, CuCl_2_(pyridazine) (JEFFOS) and CuBr_2_(pyridazine) (JEFFUY) (Thomas & Ramanan, 2016 ▸) have been, reported, but most compounds are found with Cu^I^ cations, including CuI(pyridazine) [CAQXAT (Kromp & Sheldrick, 1999 ▸) and CAQXAT01 (Thomas & Ramanan, 2016 ▸)], CuBr(pyridazine) [CAQXEX (Kromp & Sheldrick, 1999 ▸), CAQXEX01 and 02 (Thomas & Ramanan, 2016 ▸)], Cu_2_I_2_(pyridazine) (CAQXIB; Kromp & Sheldrick, 1999 ▸), Cu_2_Cl_2_(pyridazine) [CAQXOH (Kromp & Sheldrick, 1999 ▸) and CAQXOH01 and 02 (Thomas & Ramanan, 2016 ▸)], two modifications of CuCl(pyridazine) [EKINOB and EKINUH (Näther and Jess, 2003 ▸) and EKINUH01 (Thomas & Ramanan, 2016 ▸)], Cu_2_Br_2_(pyridazine) [EKIPAP (Näther & Jess, 2003 ▸) and EKIPAP01 (Thomas & Ramanan, 2016 ▸)].

## 5. Synthesis and crystallization

**Synthesis**

CdBr_2_, CdI_2_ and pyridazine were purchased from Sigma-Aldrich. All chemicals were used without further purification.

Colorless single crystals of compound **1** and **2** were obtained by the reaction of 0.500 mmol of CdBr_2_ or 0.500 mmol of CdI_2_ with 0.500 mmol of pyridazine in 1 ml of ethanol. The reaction mixtures were sealed in glass tubes and heated at 388 K for 1 d and finally cooled down to room temperature.

Larger amounts of a microcrystalline powder of **1** and **2** were obtained stirring the same amount of reactants in ethanol or water at room temperature for 1 d. For the IR spectra of **1** and **2** see Figs. S5 and S6.

**Experimental details**

The IR spectra were measured using an ATI Mattson Genesis Series FTIR Spectrometer, control software: *WINFIRST*, from ATI Mattson. The PXRD measurements were performed with Cu *K*α_1_ radiation (λ = 1.540598 Å) using a Stoe Transmission Powder Diffraction System (STADI P) equipped with a MYTHEN 1K detector and a Johansson-type Ge(111) monochromator. Thermogravimetry and differential thermoanalysis (TG-DTA) measurements were performed in a dynamic nitro­gen atmosphere in Al_2_O_3_ crucibles using a STA-PT 1000 thermobalance from Linseis. The instrument was calibrated using standard reference materials.

## 6. Refinement

Crystal data, data collection and structure refinement details are summarized in Table 3 ▸. The C-bound hydrogen atoms were positioned with idealized geometry and refined with *U*_iso_(H) = 1.2*U*_eq_(C). For compound **2**, *PLATON* (Spek, 2020 ▸) suggested a pseudo-translation along the *b*-axis with a fit of 80%. If the structure is determined in a unit cell with half of the *b*-axis, space group *Cmmm* is suggested. The structure can easily be solved in this space group but the refinement leads to only very poor reliability factors (*R*1 = 11.5%). Moreover, in this case, disorder of the nitro­gen atoms of the pyridazine ring is observed, because the N atoms of the pyridazine rings of neighboring chains are superimposed.

## Supplementary Material

Crystal structure: contains datablock(s) 1, 2. DOI: 10.1107/S2056989023002001/hb8056sup1.cif

Structure factors: contains datablock(s) 1. DOI: 10.1107/S2056989023002001/hb80561sup2.hkl

Structure factors: contains datablock(s) 2. DOI: 10.1107/S2056989023002001/hb80562sup3.hkl

Figure S1. Experimental (top) and calculated PXRD pattern (bottom) of compound 1. DOI: 10.1107/S2056989023002001/hb8056sup4.png

Figure S2. Experimental (top) and calculated PXRD pattern (bottom) of compound 2. DOI: 10.1107/S2056989023002001/hb8056sup5.png

Figure S3. DTG (top), TG (middle) and DTA curve (bottom) for compound 1. DOI: 10.1107/S2056989023002001/hb8056sup6.png

Figure S4. DTG (top), TG (middle) and DTA curve (bottom) for compound 2. DOI: 10.1107/S2056989023002001/hb8056sup7.png

Figure S5. IR spectrum for compound 1. The wave numbers of the most intense vibrations are given. DOI: 10.1107/S2056989023002001/hb8056sup8.png

Figure S6. IR spectrum for compound 2. The wave numbers of the most intense vibrations are given. DOI: 10.1107/S2056989023002001/hb8056sup9.png

CCDC references: 2245994, 2245993

Additional supporting information:  crystallographic information; 3D view; checkCIF report

## Figures and Tables

**Table 1:** Selected geometric parameters (Å, °) for **1** [Chem scheme1]

Cd1—Br1	2.7581 (2)	Cd1—N1	2.385 (2)
			
Br1^i^—Cd1—Br1^ii^	95.243 (9)	N1^i^—Cd1—Br1^ii^	91.08 (4)
Br1^i^—Cd1—Br1^iii^	84.757 (9)	N1—Cd1—Br1	88.92 (4)

Symmetry codes: (i) 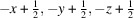 ; (ii) 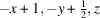 ; (iii) 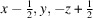 .

**Table 2:** Selected geometric parameters (Å, °) for **2** [Chem scheme1]

Cd1—I1	2.9555 (1)	Cd1—N1	2.4216 (19)
			
I1^i^—Cd1—I1^ii^	93.237 (5)	N1^i^—Cd1—I1^ii^	91.56 (4)
I1^i^—Cd1—I1^iii^	86.763 (5)	N1—Cd1—I1	88.44 (4)

Symmetry codes: (i) 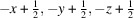 ; (ii) 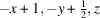 ; (iii) 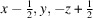 .

**Table 3:** Experimental details

	**1**	**2**
Crystal data		
Chemical formula	[CdBr_2_(C_4_H_4_N_2_)]	[CdI_2_(C_4_H_4_N_2_)]
*M*_r_	352.31	446.29
Crystal system, space group	Orthorhombic, *I* *m* *m* *a*	Orthorhombic, *I* *m* *m* *a*
Temperature (K)	293	293
*a*, *b*, *c* (Å)	7.2540 (2), 7.7223 (2), 13.2910 (4)	7.5740 (2), 8.2979 (2), 13.5363 (4)
*V* (Å^3^)	744.53 (4)	850.73 (4)
*Z*	4	4
Radiation type	Mo *K*α	Mo *K*α
μ (mm^−1^)	13.58	9.75
Crystal size (mm)	0.08 × 0.06 × 0.04	0.12 × 0.03 × 0.02

Data collection		
Diffractometer	XtaLAB Synergy, Dualflex, HyPix	XtaLAB Synergy, Dualflex, HyPix
Absorption correction	Multi-scan (*CrysAlis PRO*; Rigaku OD, 2022 ▸)	Multi-scan (*CrysAlis PRO*; Rigaku OD, 2022 ▸)
*T*_min_, *T*_max_	0.582, 1.000	0.382, 1.000
No. of measured, independent and observed [*I* > 2σ(*I*)] reflections	6952, 769, 684	7478, 889, 857
*R*_int_	0.039	0.037
(sin θ/λ)_max_ (Å^−1^)	0.772	0.774

Refinement		
*R*[*F* ^2^ > 2σ(*F* ^2^)], *wR*(*F* ^2^), *S*	0.020, 0.057, 1.16	0.016, 0.046, 1.18
No. of reflections	769	889
No. of parameters	29	30
H-atom treatment	H-atom parameters constrained	H-atom parameters constrained
Δρ_max_, Δρ_min_ (e Å^−3^)	1.07, −0.52	0.78, −0.56

Computer programs: *CrysAlis PRO* (Rigaku OD, 2022 ▸), *SHELXT2014/4* (Sheldrick, 2015*a* ▸), *SHELXL2016/6* (Sheldrick, 2015*b* ▸), *DIAMOND* (Brandenburg, 1999 ▸) and *publCIF* (Westrip, 2010 ▸).

## supplementary crystallographic information

### catena-Poly[cadmium(II)-di-µ-bromido-µ-pyridazine-κ2N1:N2] (1) Crystal data

**Table:**

[CdBr_2_(C_4_H_4_N_2_)]	*D*_x_ = 3.143 Mg m^−^^3^
*M**_r_* = 352.31	Mo *K*α radiation, λ = 0.71073 Å
Orthorhombic, *I**m**m**a*	Cell parameters from 4638 reflections
*a* = 7.2540 (2) Å	θ = 3.0–33.2°
*b* = 7.7223 (2) Å	µ = 13.58 mm^−^^1^
*c* = 13.2910 (4) Å	*T* = 293 K
*V* = 744.53 (4) Å^3^	Block, colourless
*Z* = 4	0.08 × 0.06 × 0.04 mm
*F*(000) = 640	

### catena-Poly[cadmium(II)-di-µ-bromido-µ-pyridazine-κ2N1:N2] (1) Data collection

**Table:**

XtaLAB Synergy, Dualflex, HyPix diffractometer	769 independent reflections
Radiation source: micro-focus sealed X-ray tube, PhotonJet (Mo) X-ray Source	684 reflections with *I* > 2σ(*I*)
Mirror monochromator	*R*_int_ = 0.039
Detector resolution: 10.0000 pixels mm^-1^	θ_max_ = 33.3°, θ_min_ = 3.1°
ω scans	*h* = −10→10
Absorption correction: multi-scan (CrysAlisPro; Rigaku OD, 2022)	*k* = −11→11
*T*_min_ = 0.582, *T*_max_ = 1.000	*l* = −19→20
6952 measured reflections	

### catena-Poly[cadmium(II)-di-µ-bromido-µ-pyridazine-κ2N1:N2] (1) Refinement

**Table:**

Refinement on *F*^2^	Primary atom site location: dual
Least-squares matrix: full	Hydrogen site location: inferred from neighbouring sites
*R*[*F*^2^ > 2σ(*F*^2^)] = 0.020	H-atom parameters constrained
*wR*(*F*^2^) = 0.057	*w* = 1/[σ^2^(*F*_o_^2^) + (0.0315*P*)^2^ + 0.191*P*] where *P* = (*F*_o_^2^ + 2*F*_c_^2^)/3
*S* = 1.16	(Δ/σ)_max_ < 0.001
769 reflections	Δρ_max_ = 1.07 e Å^−^^3^
29 parameters	Δρ_min_ = −0.52 e Å^−^^3^
0 restraints	

### catena-Poly[cadmium(II)-di-µ-bromido-µ-pyridazine-κ2N1:N2] (1) Special details

**Table:**

Geometry. All esds (except the esd in the dihedral angle between two l.s. planes) are estimated using the full covariance matrix. The cell esds are taken into account individually in the estimation of esds in distances, angles and torsion angles; correlations between esds in cell parameters are only used when they are defined by crystal symmetry. An approximate (isotropic) treatment of cell esds is used for estimating esds involving l.s. planes.

### catena-Poly[cadmium(II)-di-µ-bromido-µ-pyridazine-κ2N1:N2] (1) Fractional atomic coordinates and isotropic or equivalent isotropic displacement parameters (Å^2^)

**Table:**

	*x*	*y*	*z*	*U*_iso_*/*U*_eq_	
Cd1	0.250000	0.250000	0.250000	0.02829 (10)	
Br1	0.500000	0.49073 (3)	0.18013 (2)	0.03175 (10)	
N1	0.4072 (3)	0.250000	0.40756 (16)	0.0294 (4)	
C1	0.3181 (4)	0.250000	0.4947 (2)	0.0365 (6)	
H1	0.189944	0.250000	0.493718	0.044*	
C2	0.4065 (4)	0.250000	0.5870 (2)	0.0369 (6)	
H2	0.340042	0.250000	0.646880	0.044*	

### catena-Poly[cadmium(II)-di-µ-bromido-µ-pyridazine-κ2N1:N2] (1) Atomic displacement parameters (Å^2^)

**Table:**

	*U*^11^	*U*^22^	*U*^33^	*U*^12^	*U*^13^	*U*^23^
Cd1	0.01738 (14)	0.04001 (17)	0.02748 (15)	0.000	−0.00137 (9)	0.000
Br1	0.02359 (15)	0.03169 (16)	0.03996 (18)	0.000	0.000	0.00394 (10)
N1	0.0203 (10)	0.0403 (11)	0.0276 (11)	0.000	0.0005 (9)	0.000
C1	0.0216 (13)	0.0565 (18)	0.0313 (13)	0.000	0.0041 (12)	0.000
C2	0.0317 (14)	0.0527 (16)	0.0262 (12)	0.000	0.0042 (12)	0.000

### catena-Poly[cadmium(II)-di-µ-bromido-µ-pyridazine-κ2N1:N2] (1) Geometric parameters (Å, º)

**Table:**

Cd1—Br1	2.7581 (2)	N1—N1^ii^	1.346 (4)
Cd1—Br1^i^	2.7581 (2)	N1—C1	1.326 (4)
Cd1—Br1^ii^	2.7581 (2)	C1—H1	0.9300
Cd1—Br1^iii^	2.7581 (2)	C1—C2	1.385 (4)
Cd1—N1	2.385 (2)	C2—C2^ii^	1.357 (6)
Cd1—N1^iii^	2.385 (2)	C2—H2	0.9300
			
Br1—Cd1—Br1^iii^	180.0	N1^iii^—Cd1—Br1^i^	88.92 (4)
Br1^iii^—Cd1—Br1^ii^	95.243 (9)	N1—Cd1—N1^iii^	180.0
Br1^ii^—Cd1—Br1^i^	180.0	Cd1—Br1—Cd1^ii^	82.223 (7)
Br1^iii^—Cd1—Br1^i^	84.757 (9)	N1^ii^—N1—Cd1	118.58 (5)
Br1—Cd1—Br1^i^	95.244 (9)	C1—N1—Cd1	122.26 (19)
Br1—Cd1—Br1^ii^	84.756 (9)	C1—N1—N1^ii^	119.16 (16)
N1^iii^—Cd1—Br1^ii^	91.08 (4)	N1—C1—H1	118.4
N1—Cd1—Br1	88.92 (4)	N1—C1—C2	123.3 (3)
N1—Cd1—Br1^iii^	91.08 (4)	C2—C1—H1	118.4
N1^iii^—Cd1—Br1^iii^	88.92 (4)	C1—C2—H2	121.2
N1—Cd1—Br1^i^	91.08 (4)	C2^ii^—C2—C1	117.57 (17)
N1—Cd1—Br1^ii^	88.92 (4)	C2^ii^—C2—H2	121.2
N1^iii^—Cd1—Br1	91.08 (4)		

Symmetry codes: (i) *x*−1/2, *y*, −*z*+1/2; (ii) −*x*+1, −*y*+1/2, *z*; (iii) −*x*+1/2, −*y*+1/2, −*z*+1/2.

### catena-Poly[cadmium(II)-di-µ-iodido-µ-pyridazine-κ2N1:N2] (2) Crystal data

**Table:**

[CdI_2_(C_4_H_4_N_2_)]	*D*_x_ = 3.484 Mg m^−^^3^
*M**_r_* = 446.29	Mo *K*α radiation, λ = 0.71073 Å
Orthorhombic, *I**m**m**a*	Cell parameters from 6483 reflections
*a* = 7.5740 (2) Å	θ = 2.9–33.4°
*b* = 8.2979 (2) Å	µ = 9.75 mm^−^^1^
*c* = 13.5363 (4) Å	*T* = 293 K
*V* = 850.73 (4) Å^3^	Needle, colourless
*Z* = 4	0.12 × 0.03 × 0.02 mm
*F*(000) = 784	

### catena-Poly[cadmium(II)-di-µ-iodido-µ-pyridazine-κ2N1:N2] (2) Data collection

**Table:**

XtaLAB Synergy, Dualflex, HyPix diffractometer	889 independent reflections
Radiation source: micro-focus sealed X-ray tube, PhotonJet (Mo) X-ray Source	857 reflections with *I* > 2σ(*I*)
Mirror monochromator	*R*_int_ = 0.037
Detector resolution: 10.0000 pixels mm^-1^	θ_max_ = 33.4°, θ_min_ = 2.9°
ω scans	*h* = −11→11
Absorption correction: multi-scan (CrysalisPro; Rigaku OD, 2022)	*k* = −12→12
*T*_min_ = 0.382, *T*_max_ = 1.000	*l* = −20→19
7478 measured reflections	

### catena-Poly[cadmium(II)-di-µ-iodido-µ-pyridazine-κ2N1:N2] (2) Refinement

**Table:**

Refinement on *F*^2^	Hydrogen site location: inferred from neighbouring sites
Least-squares matrix: full	H-atom parameters constrained
*R*[*F*^2^ > 2σ(*F*^2^)] = 0.016	*w* = 1/[σ^2^(*F*_o_^2^) + (0.0233*P*)^2^ + 0.8677*P*] where *P* = (*F*_o_^2^ + 2*F*_c_^2^)/3
*wR*(*F*^2^) = 0.046	(Δ/σ)_max_ = 0.001
*S* = 1.18	Δρ_max_ = 0.78 e Å^−^^3^
889 reflections	Δρ_min_ = −0.56 e Å^−^^3^
30 parameters	Extinction correction: *SHELXL2016/6* (Sheldrick, 2015b), Fc^*^=kFc[1+0.001xFc^2^λ^3^/sin(2θ)]^-1/4^
0 restraints	Extinction coefficient: 0.00206 (14)
Primary atom site location: dual	

### catena-Poly[cadmium(II)-di-µ-iodido-µ-pyridazine-κ2N1:N2] (2) Special details

**Table:**

Geometry. All esds (except the esd in the dihedral angle between two l.s. planes) are estimated using the full covariance matrix. The cell esds are taken into account individually in the estimation of esds in distances, angles and torsion angles; correlations between esds in cell parameters are only used when they are defined by crystal symmetry. An approximate (isotropic) treatment of cell esds is used for estimating esds involving l.s. planes.

### catena-Poly[cadmium(II)-di-µ-iodido-µ-pyridazine-κ2N1:N2] (2) Fractional atomic coordinates and isotropic or equivalent isotropic displacement parameters (Å^2^)

**Table:**

	*x*	*y*	*z*	*U*_iso_*/*U*_eq_	
Cd1	0.250000	0.250000	0.250000	0.02666 (8)	
I1	0.500000	0.49464 (2)	0.17507 (2)	0.02931 (8)	
N1	0.4115 (3)	0.250000	0.40441 (14)	0.0272 (4)	
C1	0.3246 (4)	0.250000	0.48983 (19)	0.0354 (6)	
H1	0.201820	0.250000	0.488661	0.042*	
C2	0.4099 (4)	0.250000	0.5807 (2)	0.0377 (6)	
H2	0.346328	0.250000	0.639534	0.045*	

### catena-Poly[cadmium(II)-di-µ-iodido-µ-pyridazine-κ2N1:N2] (2) Atomic displacement parameters (Å^2^)

**Table:**

	*U*^11^	*U*^22^	*U*^33^	*U*^12^	*U*^13^	*U*^23^
Cd1	0.01738 (12)	0.03918 (15)	0.02342 (12)	0.000	−0.00147 (7)	0.000
I1	0.02338 (10)	0.02851 (11)	0.03603 (12)	0.000	0.000	0.00422 (5)
N1	0.0194 (9)	0.0420 (11)	0.0203 (8)	0.000	−0.0006 (7)	0.000
C1	0.0224 (11)	0.0607 (19)	0.0230 (10)	0.000	0.0017 (9)	0.000
C2	0.0303 (13)	0.0615 (18)	0.0212 (10)	0.000	0.0031 (10)	0.000

### catena-Poly[cadmium(II)-di-µ-iodido-µ-pyridazine-κ2N1:N2] (2) Geometric parameters (Å, º)

**Table:**

Cd1—I1	2.9555 (1)	N1—N1^ii^	1.341 (4)
Cd1—I1^i^	2.9555 (1)	N1—C1	1.330 (3)
Cd1—I1^ii^	2.9555 (1)	C1—H1	0.9300
Cd1—I1^iii^	2.9555 (1)	C1—C2	1.390 (4)
Cd1—N1	2.4216 (19)	C2—C2^ii^	1.366 (6)
Cd1—N1^iii^	2.4216 (19)	C2—H2	0.9300
			
I1^iii^—Cd1—I1	180.0	N1^iii^—Cd1—I1	91.56 (4)
I1^iii^—Cd1—I1^ii^	93.237 (5)	N1—Cd1—N1^iii^	180.0
I1^iii^—Cd1—I1^i^	86.763 (5)	Cd1—I1—Cd1^ii^	79.683 (4)
I1^i^—Cd1—I1^ii^	180.0	N1^ii^—N1—Cd1	120.33 (5)
I1^ii^—Cd1—I1	86.763 (5)	C1—N1—Cd1	120.03 (18)
I1^i^—Cd1—I1	93.237 (5)	C1—N1—N1^ii^	119.64 (16)
N1^iii^—Cd1—I1^ii^	91.56 (4)	N1—C1—H1	118.7
N1—Cd1—I1^iii^	91.56 (4)	N1—C1—C2	122.7 (3)
N1—Cd1—I1^i^	91.56 (4)	C2—C1—H1	118.7
N1^iii^—Cd1—I1^i^	88.44 (4)	C1—C2—H2	121.2
N1—Cd1—I1	88.44 (4)	C2^ii^—C2—C1	117.69 (17)
N1—Cd1—I1^ii^	88.44 (4)	C2^ii^—C2—H2	121.2
N1^iii^—Cd1—I1^iii^	88.44 (4)		

Symmetry codes: (i) *x*−1/2, *y*, −*z*+1/2; (ii) −*x*+1, −*y*+1/2, *z*; (iii) −*x*+1/2, −*y*+1/2, −*z*+1/2.
