# Supplementary figures and images for: Syntheses, crystal structures and thermal properties of catena-poly[cadmium(II)-di-μ-bromido-μ-pyridazine-κ2 N 1:N 2] and catena-poly[cadmium(II)-di-μ-iodido-μ-pyridazine-κ2 N 1:N 2]
Source: Acta Crystallogr E Crystallogr Commun. 2023 Mar 10;79(Pt 4):302–7. doi: 10.1107/S2056989023002001 (PMC10088319; doi:10.1107/S2056989023002001)

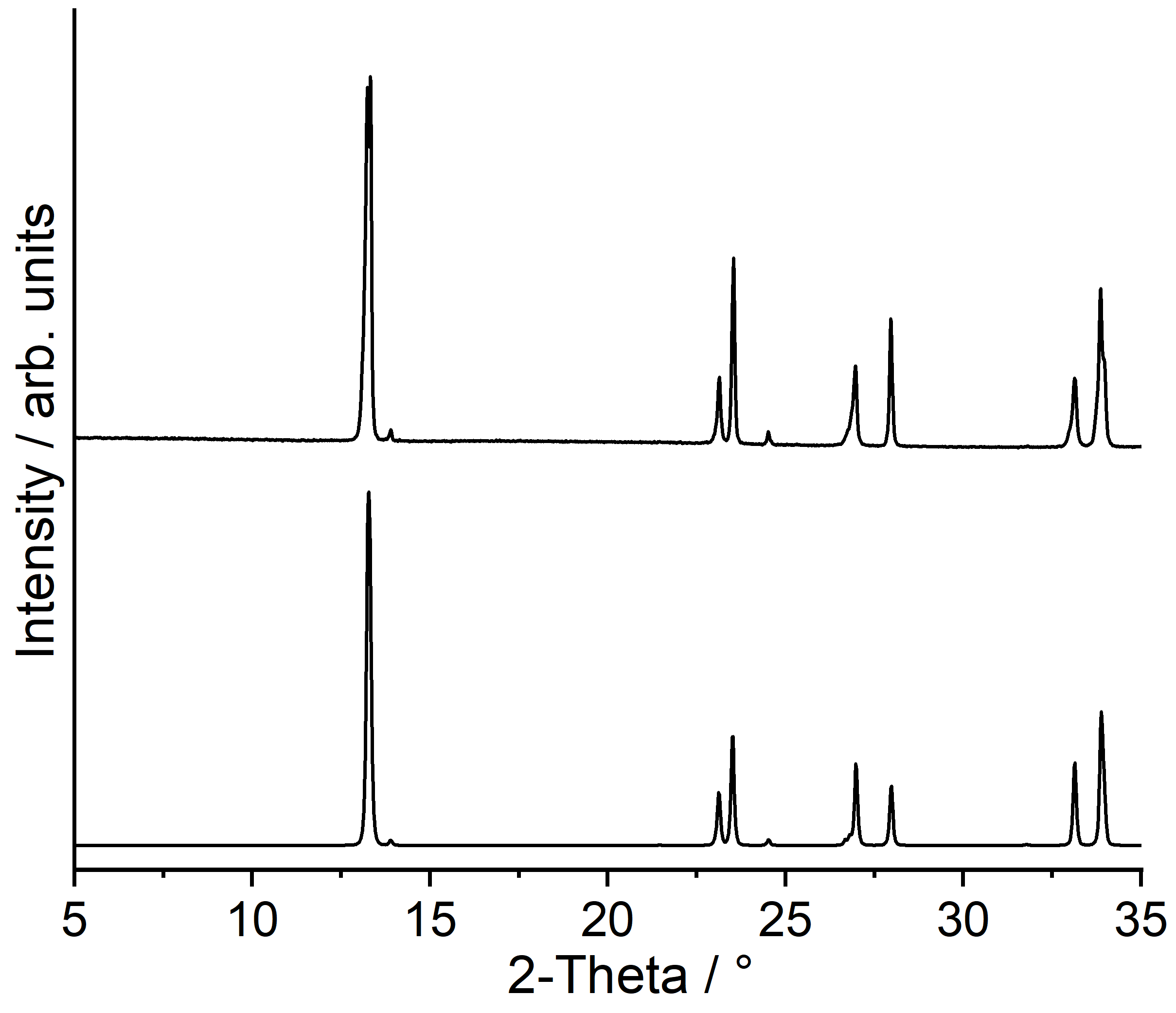

Supplement: Supplementary file 4 [file e-79-00302-sup4.png]

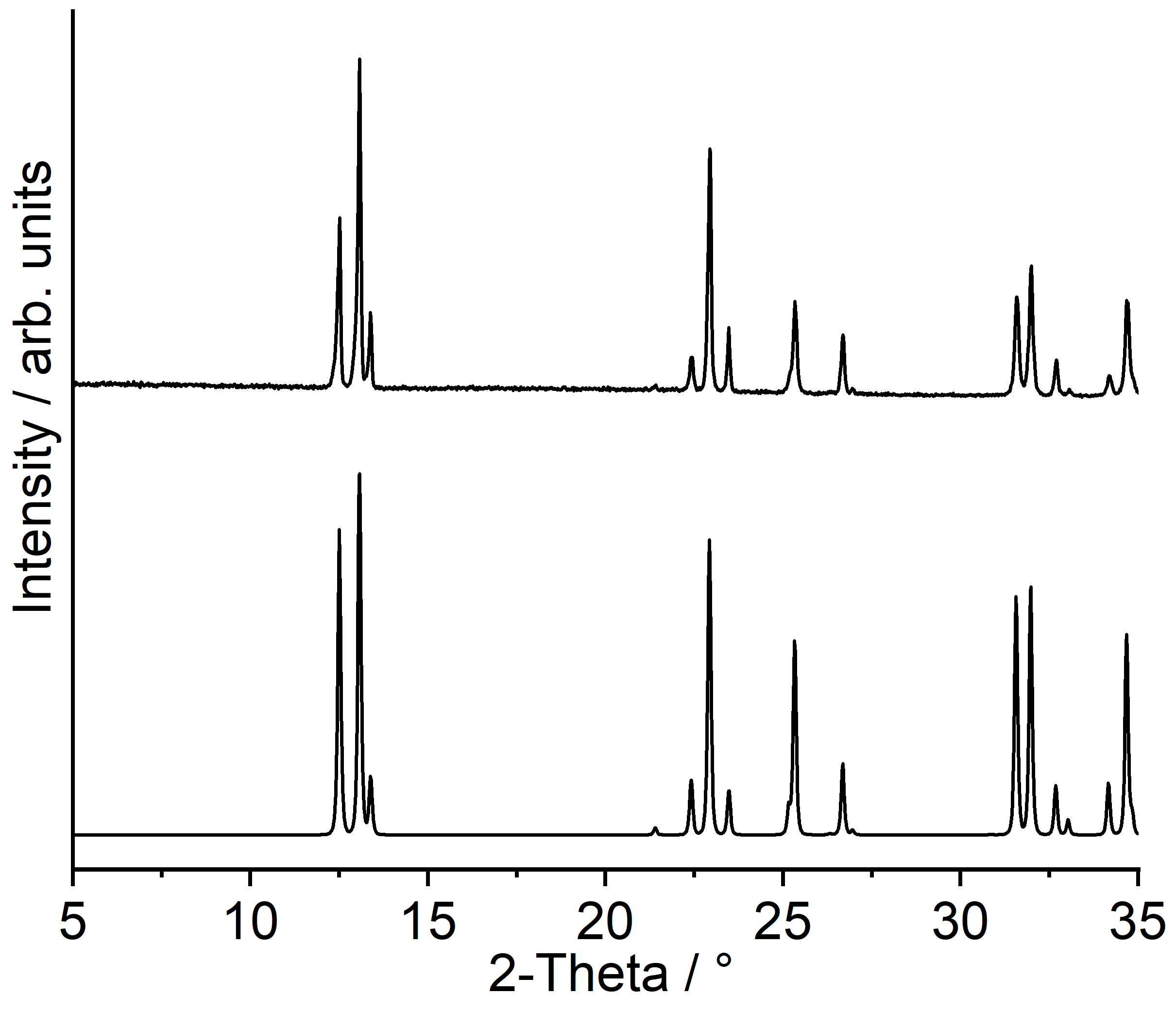

Supplement: Supplementary file 5 [file e-79-00302-sup5.png]

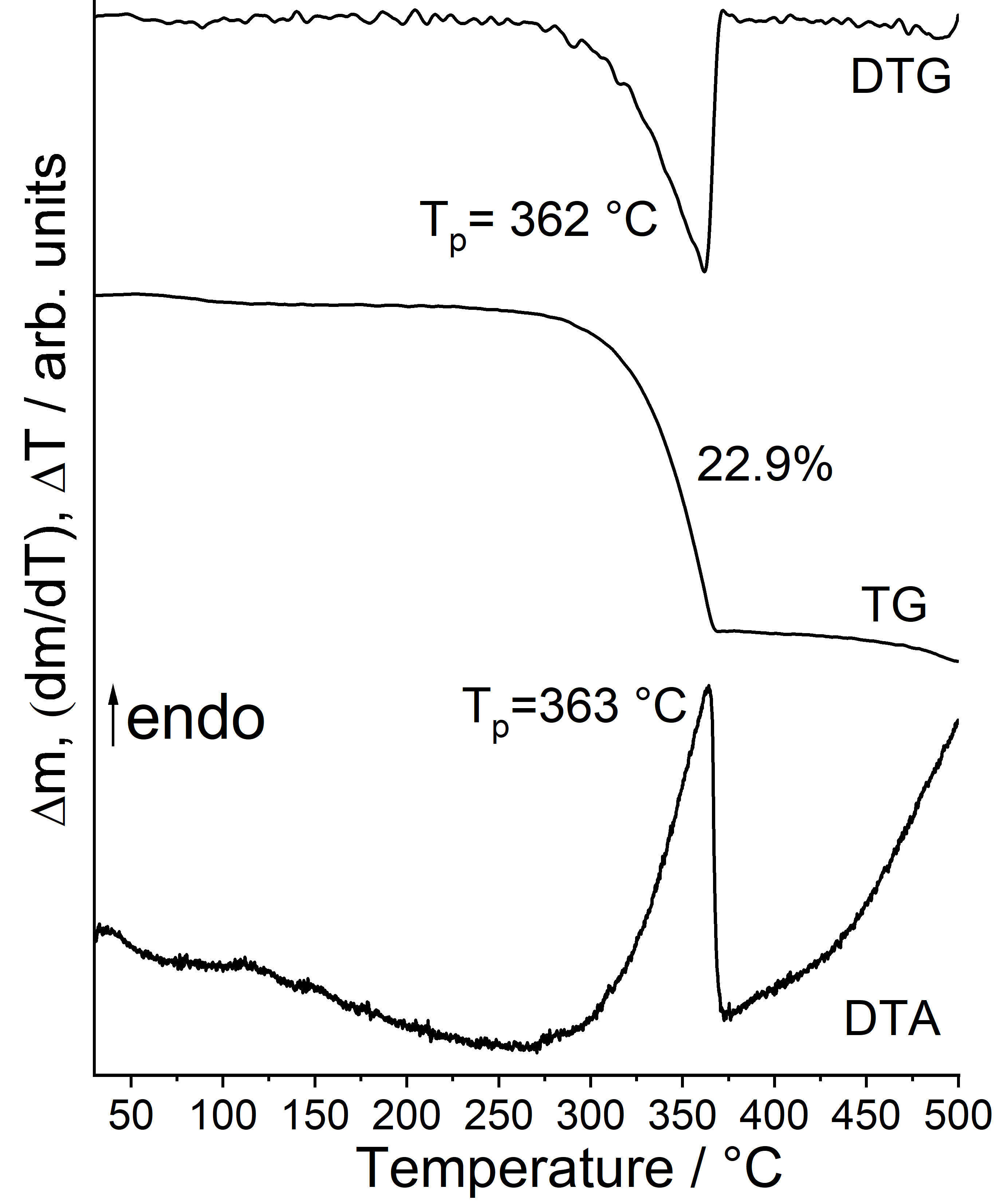

Supplement: Supplementary file 6 [file e-79-00302-sup6.png]

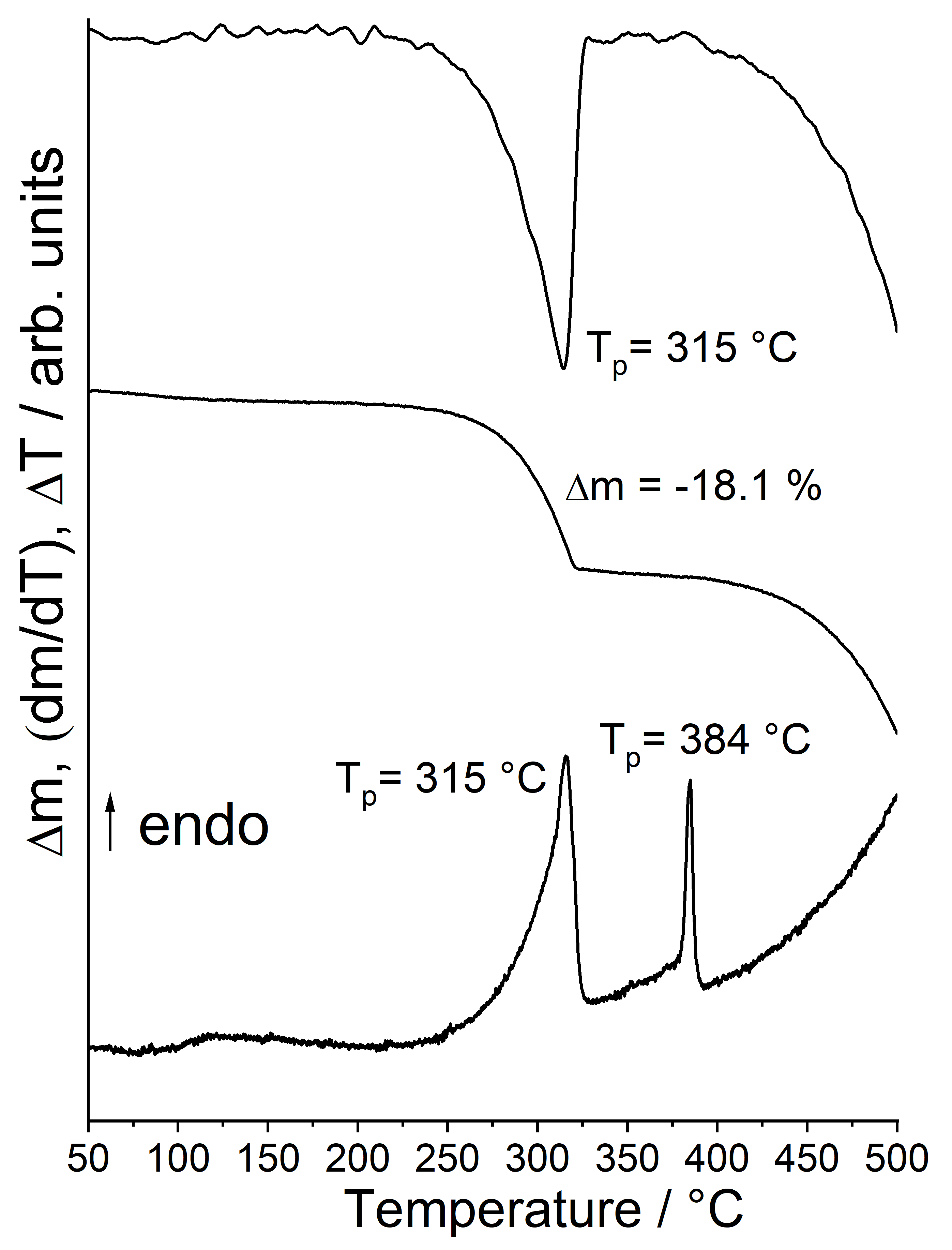

Supplement: Supplementary file 7 [file e-79-00302-sup7.png]

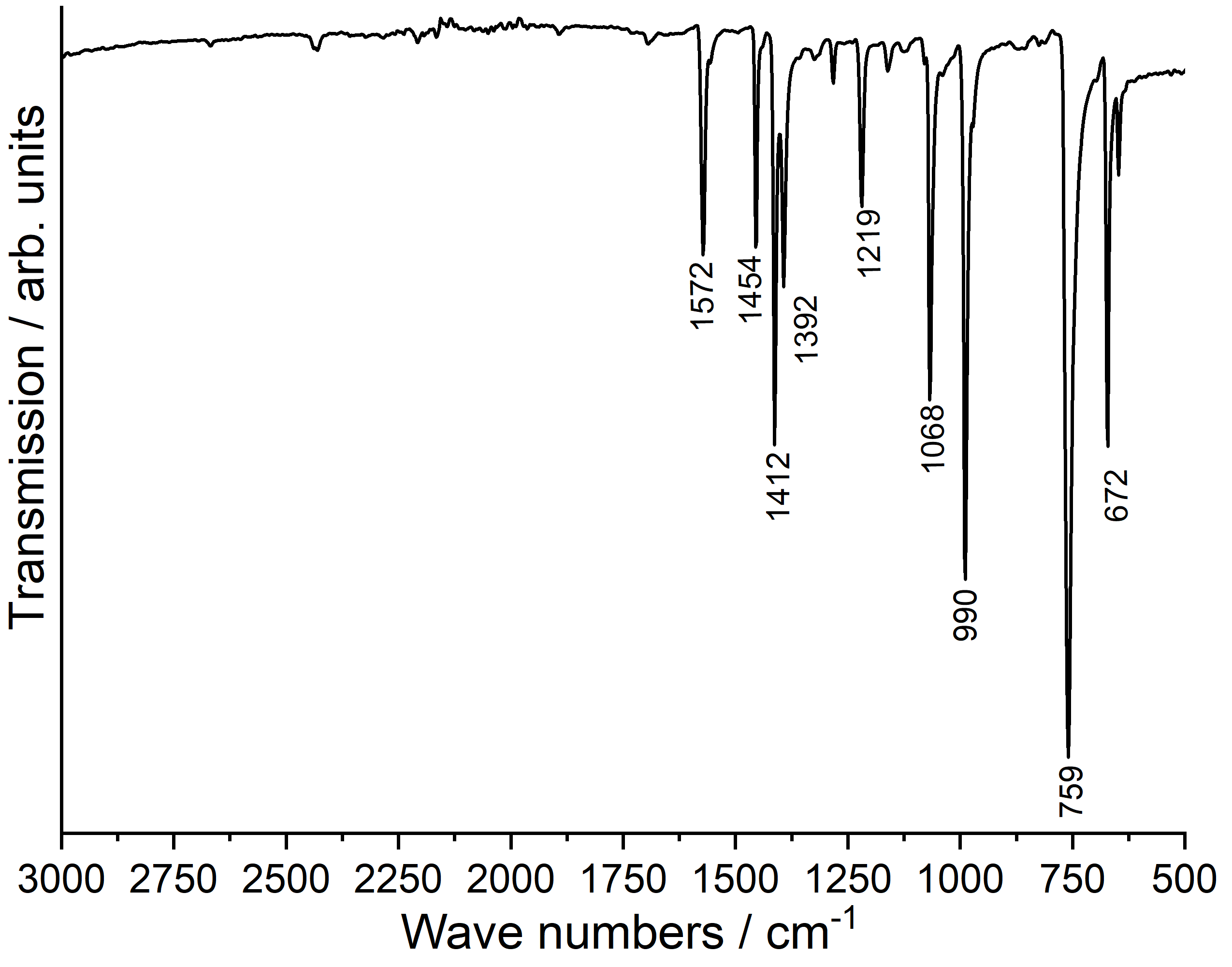

Supplement: Supplementary file 8 [file e-79-00302-sup8.png]

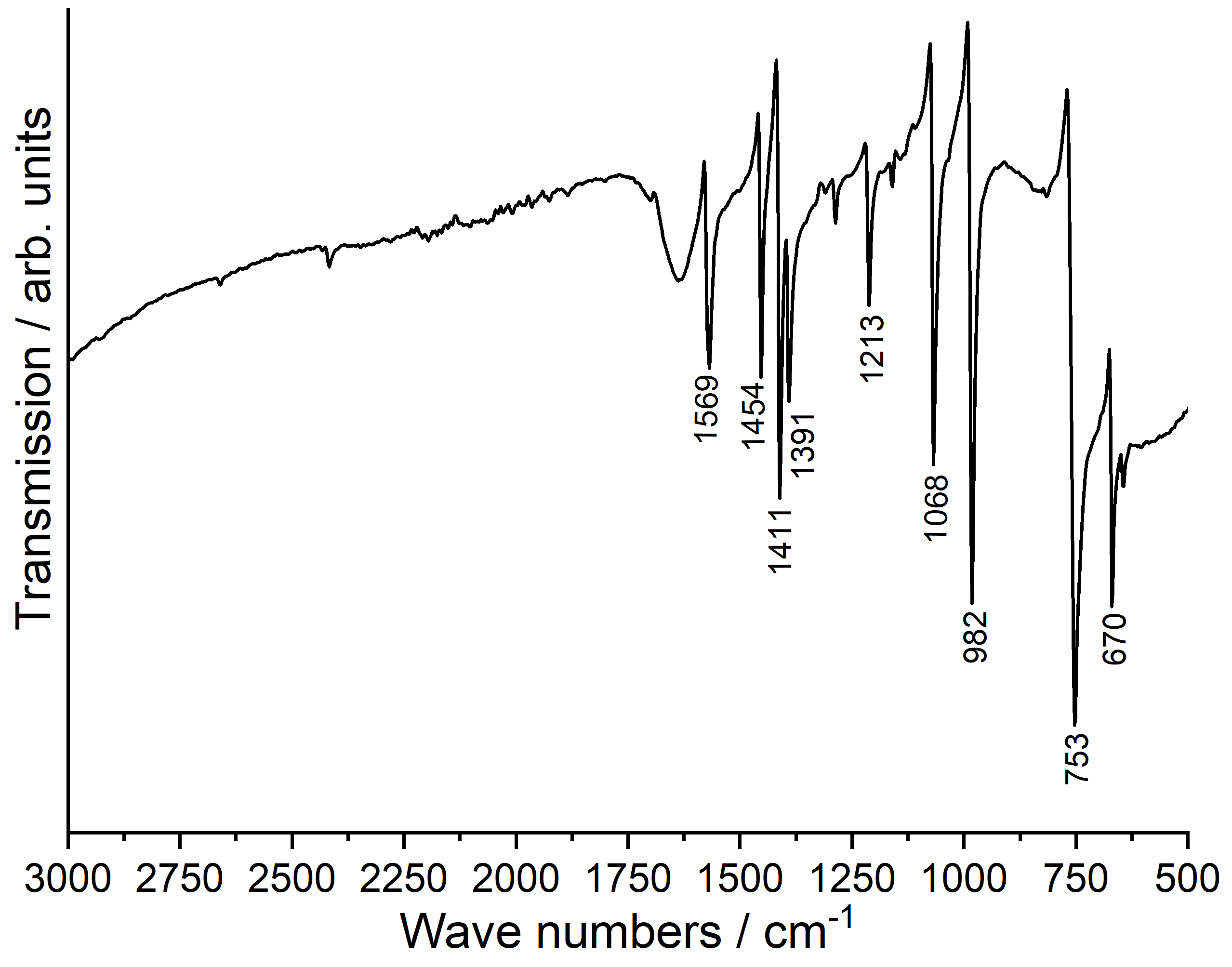

Supplement: Supplementary file 9 [file e-79-00302-sup9.png]
